# Focal adhesion kinase is activated by microtubule‐depolymerizing agents and regulates membrane blebbing in human endothelial cells

**DOI:** 10.1111/jcmm.15273

**Published:** 2020-05-26

**Authors:** Yan‐Bo Zheng, Jian‐Hua Gong, Yong‐Su Zhen

**Affiliations:** ^1^ Institute of Medicinal Biotechnology Chinese Academy of Medical Sciences and Peking Union Medical College Beijing China

**Keywords:** endothelial cells, focal adhesion kinase, IMB5046, membrane blebbing, microtubule‐depolymerizing agents, stress fibres

## Abstract

Microtubule‐depolymerizing agents can selectively disrupt tumor vessels via inducing endothelial membrane blebbing. However, the mechanism regulating blebbing is largely unknown. IMB5046 is a newly discovered microtubule‐depolymerizing agent. Here, the functions of focal adhesion kinase (FAK) during IMB5046‐induced blebbing and the relevant mechanism are studied. We found that IMB5046 induced membrane blebbing and reassembly of focal adhesions in human vascular endothelial cells. Both FAK inhibitor and knock‐down expression of FAK inhibited IMB5046‐induced blebbing. Mechanism study revealed that IMB5046 induced the activation of FAK via GEF‐H1/ Rho/ ROCK/ MLC2 pathway. cRGD peptide, a ligand of integrin, also blocked IMB5046‐induced blebbing. After activation, FAK further promoted the phosphorylation of MLC2. This positive feedback loop caused more intensive actomyosin contraction and continuous membrane blebbing. FAK inhibitor blocked membrane blebbing via inhibiting actomyosin contraction, and stimulated stress fibre formation via promoting the phosphorylation of HSP27. Conclusively, these results demonstrate that FAK is a molecular switch controlling endothelial blebbing and stress fibre formation. Our study provides a new molecular mechanism for microtubule‐depolymerizing agents to be used as vascular disrupting agents.

## 1 INTRODUCTION

In animal cells, the plasma membrane is usually associated with the underlying cortex that is rich in actin, actin‐bundling proteins and myosin.^1^, ^2^ Local detachment of the membrane from the cortex or local cortical rupture caused by the actomyosin contractions leads to spherical membrane protrusions, termed membrane blebs.^3^ Blebs have a highly dynamic life cycle that appears and disappears on a timescale of minutes. During expansion, the actin‐free membrane rapidly inflates by filling up with cytosol due to the intracellular pressure.^4^ Once expansion slows, membrane‐actin linker proteins, actin, actin‐bundling proteins and finally myosin motor proteins are recruited to the protruded membrane and drive bleb retraction.^5^ Blebbing is a common feature of cell physiology and often observed during cell movement,^6^, ^7^ apoptosis^8^ and cytokinesis.^9^, ^10^

Focal adhesions (FAs) are integrin‐based structures connecting the actin cytoskeleton to the extracellular matrix. A central component of FAs is focal adhesion kinase (FAK), an intracellular non‐receptor tyrosine kinase. Clustering of integrins promotes FAK autophosphorylation at Tyr‐397, resulting in activation of Src, which in turn leads to further FAK phosphorylation at Tyr‐576 and Tyr‐577 and facilitates its full activation.^11^ FAK is involved in FAs disassembly and cytoskeleton remodelling.^12^, ^13^ It is overexpressed and activated in many cancers and highly related with cancer growth and metastasis.^14^

Microtubule‐depolymerizing agents (MDAs) are the largest group of small molecular weight vascular disrupting agents.^15^ It has been reported that combretastatin A‐4‐phosphate (CA‐4‐P), a MDA, can induce either membrane blebbing or stress fibre formation in human endothelial cells.^16^ Endothelial blebbing will damage the vessels, although the mechanism regulating blebbing and stress fibre formation is largely unknown. IMB5046 is a novel MDA we reported previously.^17^ It shows potent cytotoxicity to tumour cell lines that are resistant to colchicine, vincristine and paclitaxel.^17^ Here, we find that IMB5046 and other MDAs induce membrane blebbing in human endothelial cells. Further study reveals that IMB5046 stimulates the activation of FAK, and FAK is a molecular switch controlling blebbing and stress fibre formation. Our study provides a new molecular mechanism for MDAs to be used as vascular disrupting agents.

## 2 MATERIALS AND METHODS

### 2.1 Antibodies and chemicals

We used the following reagents: IMB5046, Vincristine and Vinblastine from J&K Scientific Ltd.; Colchicine from SERVA Feinbiochemica; SiR‐tubulin, SiR‐actin and Rho Inhibitor I (CT04) (C3 exoenzyme covalently linked to a cell penetrating moiety) from Cytoskeleton Inc; PF‐573228 (PF‐228), PF‐431396, TAE226, Y‐27632 2HCl, Dasatinib, Bosutinib, EHop‐016, SB203580, ML141, Paclitaxel, Epothilone B and cRGD (Arg‐Gly‐Asp) peptide from Selleck Chemicals; Blebbistatin, ML‐7, ML‐9 and Nocodazole from Sigma Aldrich; all chemicals used were of analytical grade. Following antibodies were used: p‐FAK(Tyr397)(D20B1), FAK(D2R2E), p‐MLC(Ser19)(3675), MLC(D18E2), p‐HSP27(Ser82)(D1H2F6), HSP27(D6W5V) and GEF‐H1(55B6) from Cell Signaling Technology; β‐Actin(TA‐09), FITC‐conjugated anti‐Mouse IgG, HRP‐conjugated anti‐Mouse IgG and HRP‐conjugated anti‐Rabbit IgG from Zhongshan Golden Bridge Biotechnology.

### 2.2 Cell cultures

Human microvascular endothelial cell (HMEC‐1) was cultured at 37°C in Dulbecco's modified Eagle's medium (DMEM) (Hyclone) supplemented with 10% foetal bovine serum, 2 mM glutamine, 100 μg/mL streptomycin and 100 U/mL penicillin in a humidified atmosphere containing 5% CO_2_. Human umbilical vein endothelial cells (HUVECs, pooled donors) were purchased from Lonza and cultured in EGM^TM^‐2 medium containing EGM™‐2 SingleQuots™ kit in a humidified atmosphere containing 5% CO_2_.

### 2.3 Live‐cell imaging

HMEC‐1 cells were seeded in 4‐chamber 35 mm glass bottom dish with 20 mm micro‐well (Cellvis and grown to 80%‐confluence. Then, 100 nM SiR‐tubulin and SiR‐actin were added for 6‐12 hours. Videos were acquired using an Olympus IX81 fluorescence microscopy equipped with a heating stage heated to 37°C. The frame interval is indicated in the supplementary movie legends.

### 2.4 Immunofluorescence assay

Cells grown on coverslips were fixed in 4% paraformaldehyde, permeabilized with 0.1% Triton X‐100 and blocked with 5% bovine serum albumin. Cells were then incubated with primary antibodies (FAK, p‐FAK, and p‐MLC2) for 1 hour, washed thrice and incubated with secondary antibodies for another hour. For F‐actin staining, the fixed cells were stained with 50 μg/mL phalloidin‐FITC (Sigma Aldrich) for 40 minutes. For vinculin staining, the fixed cells were stained with eFluor 570‐labelled vinculin antibody (7F9) (eBioscience) for 1 hour. The images were observed and collected using an Olympus IX81 fluorescence microscopy or a ZEISS LSM 710 laser scanning confocal microscopy. Diameter of blebs and area of FAs were measured using ImageJ software (version 1.49, National Institutes of Health).

### 2.5 SDS‐PAGE and Western blot assay

Cells were lysed in the lysis buffer (50 mM Tris‐HCl, pH8.0, 150 mM NaCl, 0.02% NaN3, 0.1% SDS, 100 μg/mL PMSF, 1 μg/mL aprotinin, 1% NP‐40, 0.5% sodium deoxycholate). Protein concentration was quantified using a BCA protein assay kit (Thermo Scientific). Equal amounts of protein (20‐50 μg/lane) were separated on SDS‐PAGE and transferred to PVDF membrane. After blocked with 2% bovine serum albumin, the membrane was incubated with primary antibody and HRP‐conjugated secondary antibody, sequentially. The immunoreactive bands were visualized using the Immobilion Western Chemiluminescent HRP Substrate kit (Millipore), and the images were captured using FluorChem HD2 imaging system (ProteinSimple). Band density was measured with ImageJ software. Then, the values were corrected by the density of β‐Actin or total (phosphorylated and unphosphorylated) protein and normalized to the control expressed as 1. Western blot analysis was repeated at least three times.

### 2.6 Rho pull‐down assay

Rho activation was measured using Active Rho detection kit (Cell Signaling Technology) following the manufacturer's instructions. Briefly, HMEC‐1 cells were seeded in 75 cm^2^ flask to grow to a 70%‐80% confluence and treated with 1 μM IMB5046 for 30 minutes, then exposed to 10 μM PF‐228 or not for 10 minutes. Subsequently, cells were lysed and incubated with GST‐Rhotekin‐RBD and glutathione resin to selectively isolate and pull down the active form of Rho. The active form was detected by immunoblotting using a Rho antibody (8789, Cell Signaling Technology). Densitometric values of immunoblots were obtained using ImageJ. The values were corrected by the density of total Rho and normalized to the control expressed as 1. The experiment was repeated three times.

### 2.7 RNA interference

HMEC‐1 cells at 80% confluence were harvested and seeded in 6‐well plate at 50 000 cells/well in DMEM containing 10% FBS. Ten μL of 10 μM FAK siRNA (Cell Signaling Technology, 5 μL of 20 μM GEF‐H1 siRNA (target sequence: GCTGCAGAATCTAATCGTA) or negative control siRNA (RiboBio Co.), 12 μL riboFECT™ CP Reagent and 120 μL riboFECT™ CP Buffer were mixed, and cells were transfected according to manufacturer's protocols (RiboBio Co.). After 96 hours, total proteins were extracted and analysed by Western blot. Three independent experiments were performed. For staining of F‐actin, 12 500 cells were seeded in 4‐chamber 35‐mm glass bottom dish with 20 mm micro‐well and transfected with siRNA as above. After 72 hours, cells were fixed and stained with phalloidin‐FITC.

### 2.8 Statistical analysis

All statistical analyses were done using Prism GraphPad software v5.0. Results were depicted as mean ± SD. An unpaired two‐tailed Student's *t* test was performed for statistical comparison, and *P*‐values < .05 were considered statistically significant.

## 3 RESULTS

### 3.1 IMB5046 induces reassembly of cytoskeleton and membrane blebbing in human endothelial cells

**Figure 1:**
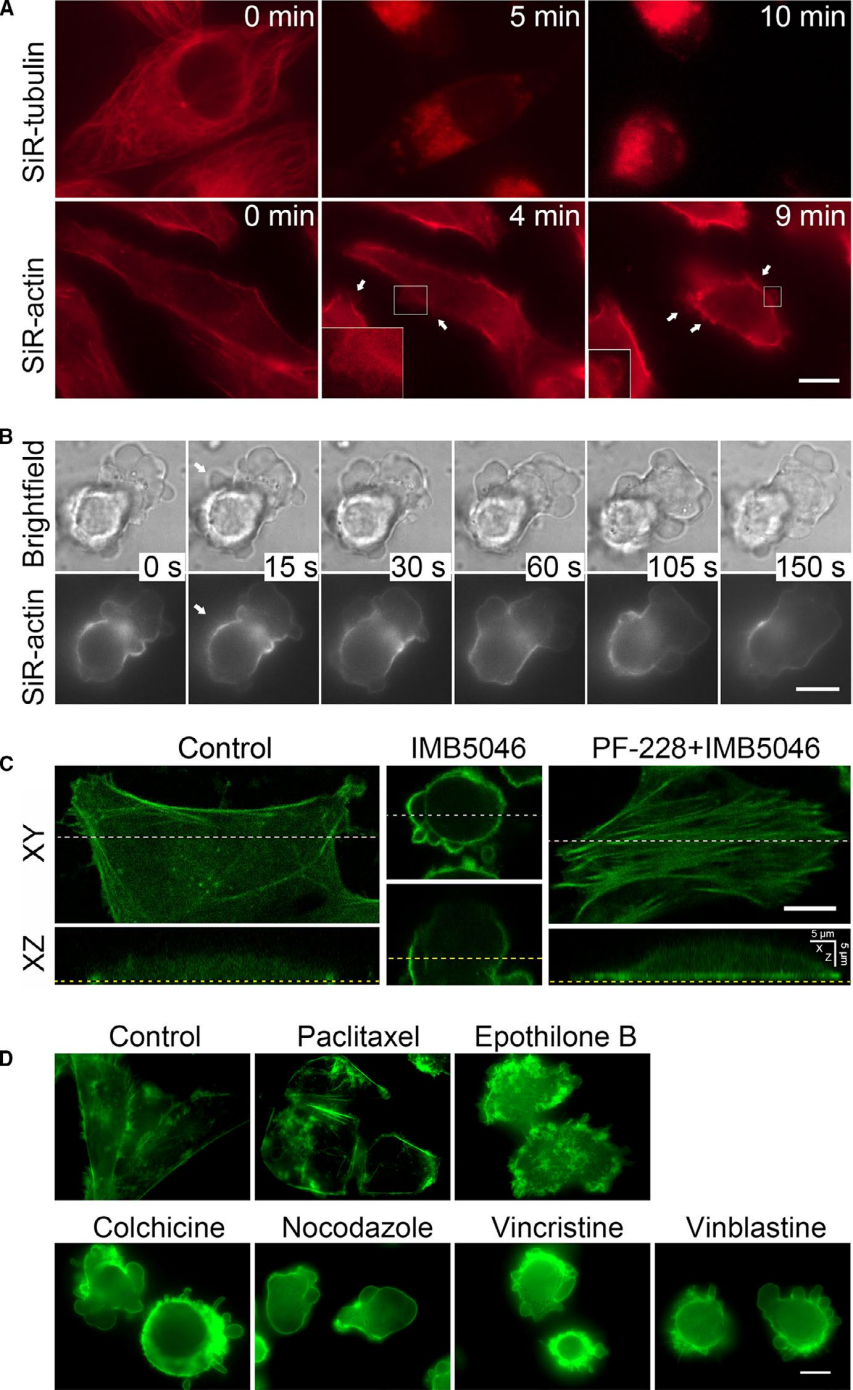
IMB5046 induces reassembly of cytoskeleton and endothelial blebbing. A, Effects of IMB5046 on cytoskeleton. HMEC‐1cells were labelled with SiR‐tubulin or SiR‐actin, then exposed to 1 μM IMB5046. Timing relative to IMB5046 exposure is indicated in white letters. Blebs are indicated by the arrows. Boxed regions show enlarged blebs. Bar, 10 μm. B, Life time of IMB5046‐induced blebs. HMEC‐1 cells were labelled with SiR‐actin and treated with 1 μM IMB5046 for 30 minutes. Timing relative to the first image is indicated. The separation locus of membrane from the actin cortex is indicated by the arrows. Bar, 10 μm. C, IMB5046 and PF‐228 induce reorganization of actin cytoskeleton. HMEC‐1 cells were pre‐treated with PF‐228 (10 μM, 30 minutes) or not, then exposed to 1 μM IMB5046 for 1 hour and stained with phalloidin‐FITC. XZ‐sections were generated from confocal Z‐stacks along the white broken line (Top panel). XY‐sections were generated along the yellow broken line (Bottom panel). Bar, 10 μm. D, Effects of different microtubule inhibitors on membrane blebbing. HMEC‐1 cells were treated with different microtubule inhibitors (1 μM) for 1 hour, then stained with phalloidin‐FITC. Bar, 10 μm

As a newly discovered MDA, we first investigated the effects of IMB5046 on cytoskeleton of HMEC‐1 cells using live‐cell imaging. IMB5046 led to cell contraction and disrupted microtubule structure within 5 minutes (Figure 1A). About 2‐10 minutes later, the cells came to bleb (Figure 1A; Movie S1) and lasted for 5‐6 hours. The blebs expanded about 30 seconds, then retracted about 2 minutes (Figure 1B). We also observed the detachment of the membrane from the actin cortex (Figure 1B). As 1 μM IMB5046 induced intensive blebbing in about 94.5% of cells in 30 minutes, this concentration was used in the following experiments.

Then, the effects of IMB5046 on actin cortex were observed using laser scanning confocal microscopy. In control cells, F‐actin was distributed at the cell periphery with few filaments traversing the cells, and just a very weak fluorescence was observed at the dorsal side of the cell in XZ‐section (Figure 1C). Whereas, in IMB5046‐treated cells, a strong cortical staining was observed at the dorsal side, especially in blebs (Figure 1C), and cell height was increased from 8.97 ± 1.17 μm to 16.64 ± 3.39 μm (20 cells).

Further experiments showed that IMB5046 could induce blebbing of human umbilical vein endothelial cells HUVEC (Figure S1A), but not of murine embryonic fibroblast cells NIH/3T3 or human lung carcinoma cells NCI‐H460 (data not shown).

The effect of other microtubule inhibitors on endothelial blebbing was also studied. Figure 1D showed that all of the agents including microtubule‐stabilizing agents (paclitaxel and epothilone B) and MDAs (colchicine, nocodazole, vincristine and vinblastine) induced cell contraction. Paclitaxel caused a strong staining of F‐actin at the cell periphery, and epothilone B induced F‐actin–rich membrane ruffles, whereas either agent did not induce blebbing (Figure 1D). In contrast, all of the tested MDAs induced blebbing which resembled the appearance induced by IMB5046 (Figure 1D).

### 3.2 IMB5046 induces reassembly of FAs, and FAK activity is required for membrane blebbing

**Figure 2:**
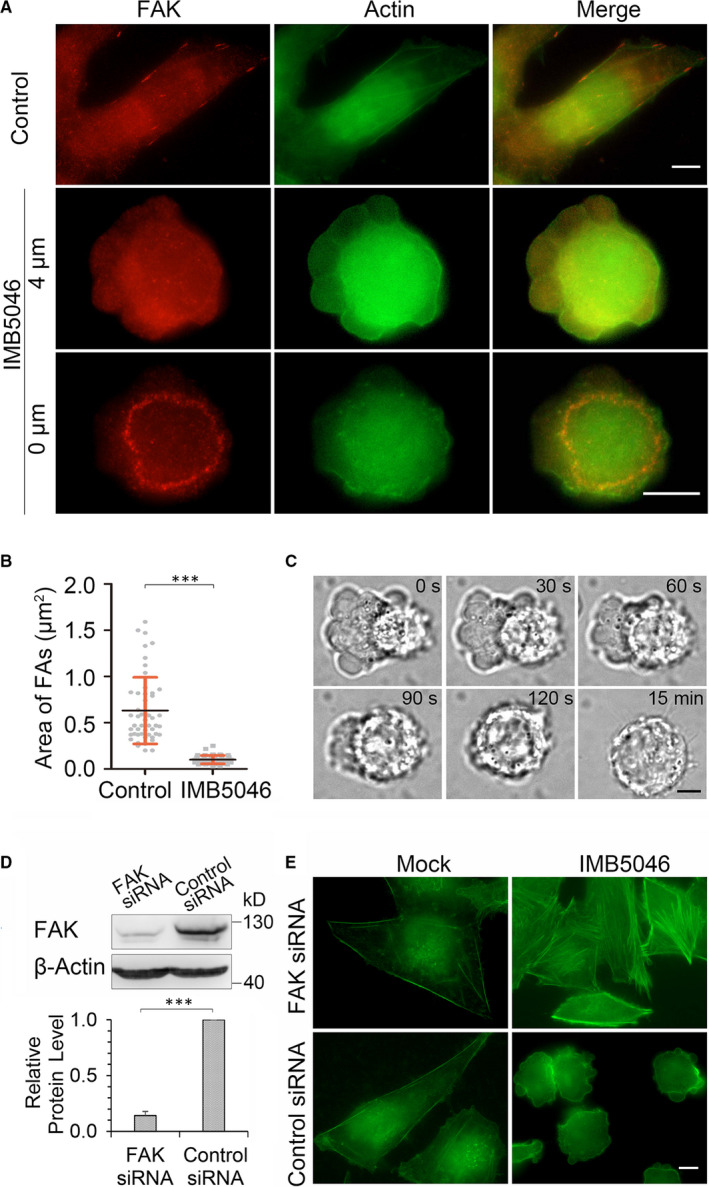
IMB5046 induces reassembly of FAs, and FAK activity is required for blebbing. (A, B) Distribution pattern of FAs after IMB5046 treatment. HMEC‐1 cells were exposed to 1 μM IMB5046 for 1 hour, then stained with FAK antibody and phalloidin‐FITC. 4 μm, focal plane 4 μm above the bottom. 0 μm, focal plane 0 μm above the bottom. Bar, 10 μm. The area of FAs was calculated according to FAK staining. Data are presented as mean ± SD (n = 50). C, Time‐lapse images of PF‐228 inhibiting blebbing. HMEC‐1 cells were treated with 1 μM IMB5046 for 1 hour; then, 10 μM PF‐228 was added. Timing relative to IMB5046 exposure is indicated. Bar, 5 μm. (D, E) Knock‐down expression of FAK blocks IMB5046‐induced blebbing. HMEC‐1 cells were exposed to FAK siRNA or control siRNA for 72 hours; then, the expression level of FAK was detected by Western blot. The histogram shows the relative protein level of FAK. Data are presented as mean ± SD (n = 3). For fluorescence assay, after knock‐down expression of FAK, cells were treated with IMB5046 (1 μM, 1 hour) or not, then stained with phalloidin‐FITC. Bar, 10 μm. ***P < .001

FAs and actin cytoskeleton are physically coupled and functional interdependent structures.^18^ Further experiment was performed to verify whether IMB5046 induces reassembly of FAs. As shown in Figure 2A, in control cells, FAK distributed at the end of the stress fibres at the periphery or centre of the cells. After treatment with IMB5046, FAs displayed a circular or oval distribution pattern along the cell periphery (Figure 2A) and partially co‐localized with actin cortex at the ventral side of the cells (Figure S2). It indicates that FAs are associated with actin cortex. Quantification of the presence of FAK showed that the size of FAs in IMB5046‐treated cells decreased about 6 times compared with that in control cells (Figure 2B). When vinculin antibody was used, similar result was obtained (Figure S3). In HUVEC cells, IMB5046 also induced similar distribution pattern changes of FAs (Figure S1A). All of those results suggest that IMB5046 induces reassembly of FAs.

FAK regulates the formation, disassembly and/or maturation of FAs.^12^, ^19^ We next investigated whether inhibiting the activity of FAK could block IMB5046‐induced blebbing. HMEC‐1 cells were treated with IMB5046 to induce blebbing; then, FAK inhibitor PF‐228 was added. Live‐cell imaging showed that from the onset of PF‐228 exposure, the blebs came to retract. About 2‐3 minutes later, PF‐228 blocked blebbing in about 80% cells (Figure 2C; Movie S2). Though some cells were still blebbing, the blebs became smaller in size and less in amount. This result was also confirmed in HUVEC cells (Figure S1B). We further detected the effects of some other FAK inhibitors on blebbing and found that both PF‐431396 and TAE226 blocked IMB5046‐induced blebbing (Table S1). As a downstream effector of FAK, Src inhibitor dasatinib and bosutinib also inhibited blebbing (Table S1).

To further demonstrate the effects of FAK on blebbing, we used siRNA to knock down the expression of FAK (Figure 2D). The result showed that IMB5046 failed to induce membrane blebbing in these cells. On the contrary, it stimulated the formation of large numbers of stress fibres in the cytoplasm (Figure 2E).

### 3.3 FAK is activated via GEF‐H1/ Rho/ ROCK/ MLC2/ Integrin pathway

**Figure 3:**
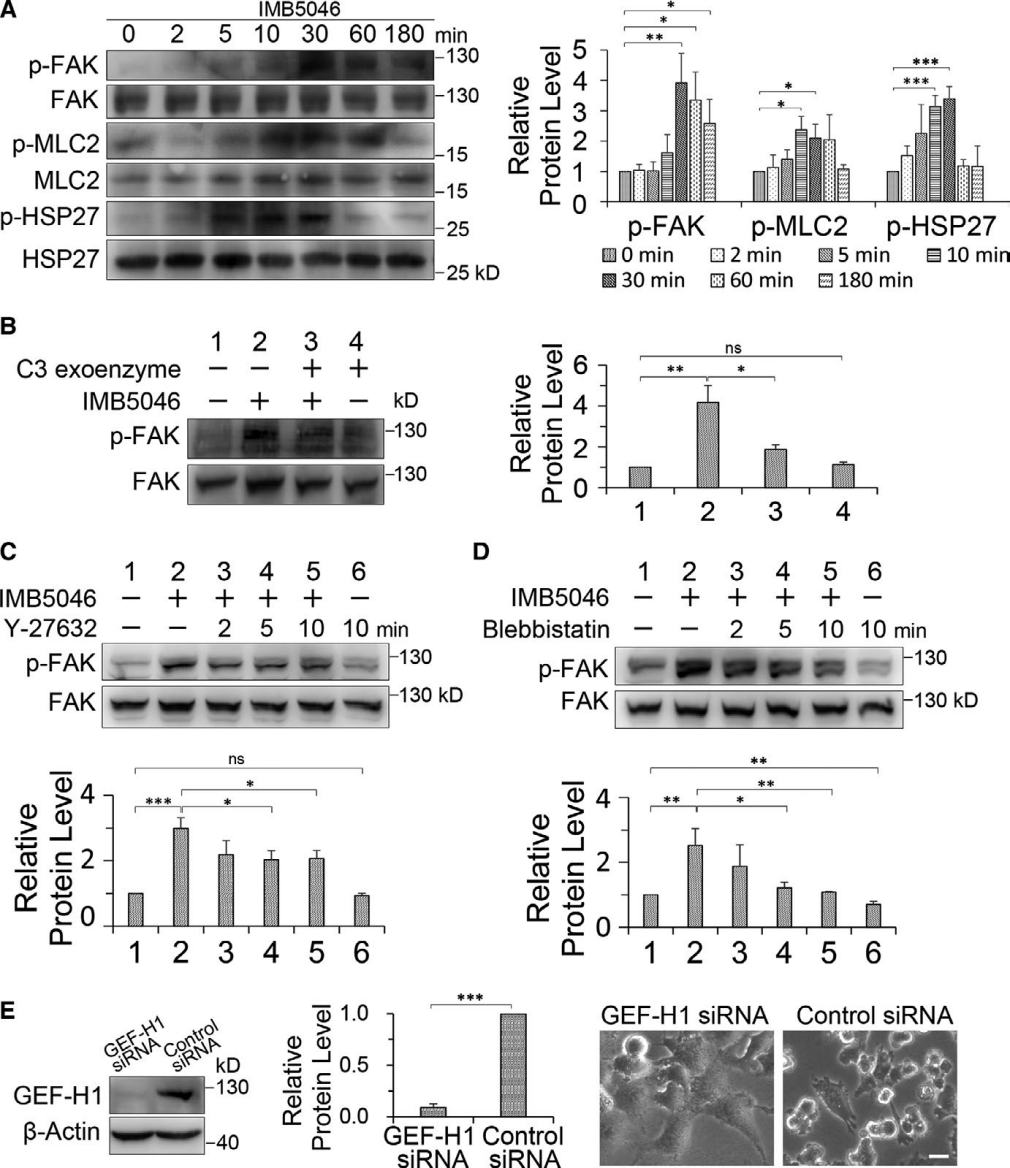
FAK is activated via GEF‐H1/ Rho/ ROCK/ MLC2 pathway. A, IMB5046 stimulates phosphorylation of FAK, MLC2 and HSP27. Representative images of Western blot are shown. The histogram shows the relative protein level. B, C3 exoenzyme inhibits IMB5046‐induced activation of FAK. HMEC‐1 cells were pre‐treated with C3 exoenyme (1 μg/mL, 6 hours) or not, then exposed to 1 μM IMB5046 for 1 hour. Representative images of Western blot are shown. The histogram shows the relative protein level of p‐FAK. (C, D) Regulation of FAK activity by ROCK and myosin. HMEC‐1 cells were treated with 1 μM IMB5046 for 1 hour; then, 10 μM Y‐27632 or blebbistatin was added for different time. Representative images of Western blot are shown. The histogram shows the relative protein level of p‐FAK. E, Knock‐down expression of GEF‐H1 inhibits IMB5046‐induced blebbing. HMEC‐1 cells were exposed to GEF‐H1 siRNA or control siRNA for 72 hours, then exposed to 1 μM IMB5046 for 1 hour and photographed. The expression level of GEF‐H1 was detected by Western blot. The histogram shows the relative protein level of GEF‐H1. Bar, 20 μm. All data are presented as mean ± SD (n = 3). ***P < .001. **P < .01. *P < .05. ns, no significance

The activation of FAK was verified by Western blot. HMEC‐1 cells were treated with 1 μM IMB5046 for different time; then, the phosphorylation level of FAK at Tyr‐397 was detected. The result showed that IMB5046 induced the phosphorylation of FAK. The maximum phosphorylation was observed at about 30 minutes after IMB5046 treatment and lasted at least 3 hours (Figure 3A). IMB5046 also promoted the phosphorylation of myosin regulatory light chain (MLC2) and small heat shock protein HSP27 (Figure 3A). p‐MLC2 can stimulate the myosin contraction, and p‐HSP27 can promote the actin polymerization.^20^

**Figure 4:**
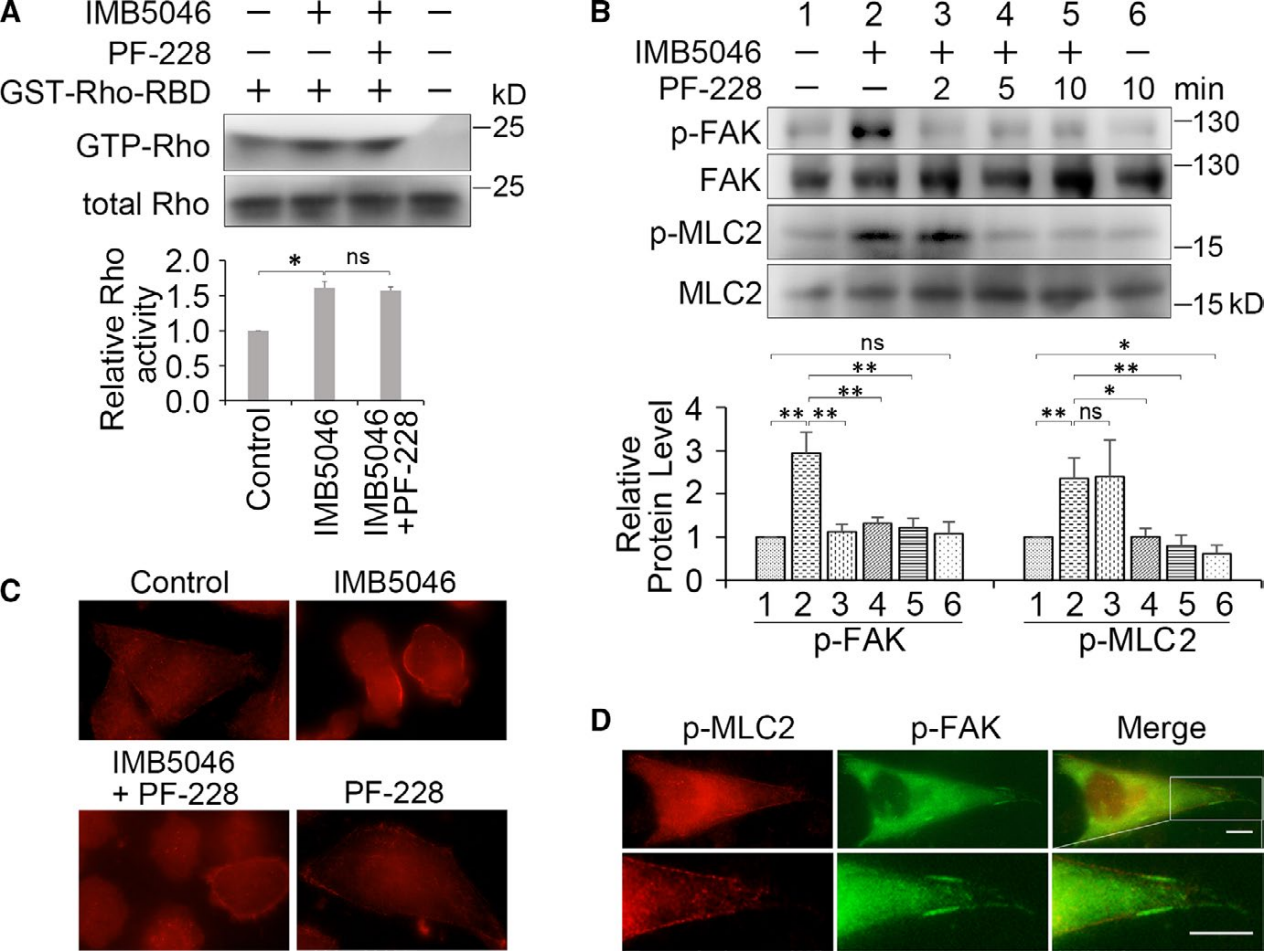
FAK inhibitor blocks blebbing via inhibiting the phosphorylation of MLC2. A, Rho activity assay. HMEC‐1 cells were treated with 1 μM IMB5046 for 30 minutes, then exposed to 10 μM PF‐228 or not for 10 minutes. The histogram shows the relative level of active Rho. B, PF‐228 inhibits the phosphorylation of FAK and MLC2. HMEC‐1 cells were treated with 1 μM IMB5046 for 1 hour, then exposed to 10 μM PF‐228 for different time. Representative images of Western blot are shown. The histogram shows the relative protein level of p‐FAK and p‐MLC2. C, Effects of IMB5046 and PF‐228 on p‐MLC2. HMEC‐1 cells were treated with 1 μM IMB5046 for 1 hour, then exposed to 10 μM PF‐228 for 10 minutes. Cells were stained with p‐MLC2 antibody. Bar, 10 μm. D, Co‐localization of p‐MLC2 and p‐FAK. HMEC‐1 cells were stained with p‐MLC2 and p‐FAK antibody. High‐magnification image of the boxed area is shown in the bottom panel. Bar, 10 μm. All data are presented as mean ± SD (n = 3). ***P < .001. **P < .01. *P < .05. ns, no significance

Rho family of small guanosine triphosphatases (GTPases) regulate the assembly of actin cytoskeleton and cell adhesion.^21^ The best‐studied Rho GTPases regulating cell adhesion are Rho, Rac and Cdc42.^22^ We first investigated the effects of different Rho GTPases inhibitors on blebbing. The results showed that Rac inhibitor EHop‐016 and CDC42 inhibitor ML141 showed no effects on blebbing (Table S1), whereas Rho inhibitor C3 exoenzyme and its downstream Rho‐associated kinase (ROCK) inhibitor Y‐27632 obviously blocked blebbing (Table S1). Rho activity assay showed that IMB5046 stimulated the activation of Rho (Figure 4A). It indicates that IMB5046 activates Rho/ROCK pathway, and this pathway is required for blebbing. We also found that myosin Ⅱ inhibitor blebbistatin inhibited blebbing (Table S1). It further verifies that myosin contraction is required for blebbing. As MLCK (myosin light chain kinase) inhibitor ML‐7 and ML‐9 could not inhibit blebbing (Table S1) and ROCK could phosphorylate MLC2 directly and/or inhibit myosin phosphatase, ^23^ we propose that MLC2 is phosphorylated via Rho/ROCK pathway.

Further experiments focus on whether Rho/ ROCK/ MLC2 pathway is involved in the activation of FAK. Western blot showed that both C3 exoenzyme and Y‐27632 blocked IMB5046‐induced phosphorylation of FAK (Figure 3B, C). When blebbistatin was used, similar result was observed (Figure 3D). It demonstrates that both Rho/ROCK pathway and myosin contraction are required for FAK activation. Actomyosin contraction would cause integrin clustering and finally stimulate the autophosphorylation of FAK.^24^ To verify the role of integrin during blebbing, cRGD peptide, a ligand of integrin, which can block the interaction between integrin and extracellular matrix, was used. The result showed that cRGD peptide blocked IMB5046‐induced blebbing (Table S1).

Rho GTPases are activated by guanine nucleotide exchange factors (GEFs) that stimulate the exchange of bound GDP for GTP. It has been reported that GEF‐H1 can bind with microtubule and release of it activates Rho.^25^, ^26^ To investigate the role of GEF‐H1 during blebbing, we knocked down the expression of GEF‐H1 by RNAi and found that down‐regulation of GEF‐H1 blocked IMB5046‐induced blebbing completely (Figure 3E). Whereas in control siRNA treated cells, IMB5046 induced blebbing in about 83.5% cells.

All of these results demonstrate that IMB5046 disrupts microtubule, resulting in release of GEF‐H1, which in turn activates Rho/ROCK pathway, leads to phosphorylation of MLC2 and actomyosin contraction and finally induces integrin clustering and autophosphorylation of FAK.

### 3.4 FAK inhibitor blocks blebbing via inhibiting the phosphorylation of MLC2

The mechanism of PF‐228 blocking blebbing was investigated. Rho activity assay indicated that PF‐228 showed no obvious effect on the activity of Rho (Figure 4A). As myosin contraction plays an essential role during blebbing, we detected the effect of PF‐228 on the phosphorylation of MLC2. The result showed that PF‐228 induced the down‐regulation of p‐FAK in 2 minutes and down‐regulation of p‐MLC2 in 5 minutes (Figure 4B). Down‐regulation of MLC2 was further confirmed using IIF assay. Figure 4C showed that in IMB5046‐treated cells, enhanced red fluorescence of p‐MLC2 was observed, especially in the cortex of blebs. Whereas in IMB5046 plus PF‐228 treated cells, just a weak staining of p‐MLC2 was observed. PF‐228 alone showed no obvious effect on p‐MLC2. These results demonstrate that PF‐228 blocks blebbing via inhibiting the actomyosin contraction. Our result also showed that p‐MLC2 and p‐FAK co‐localized at the FA sites (Figure 4D).

### 3.5 IMB5046 stimulates the formation of stress fibres in cells pre‐treated with PF‐228 via promoting the phosphorylation of HSP27

**Figure 5:**
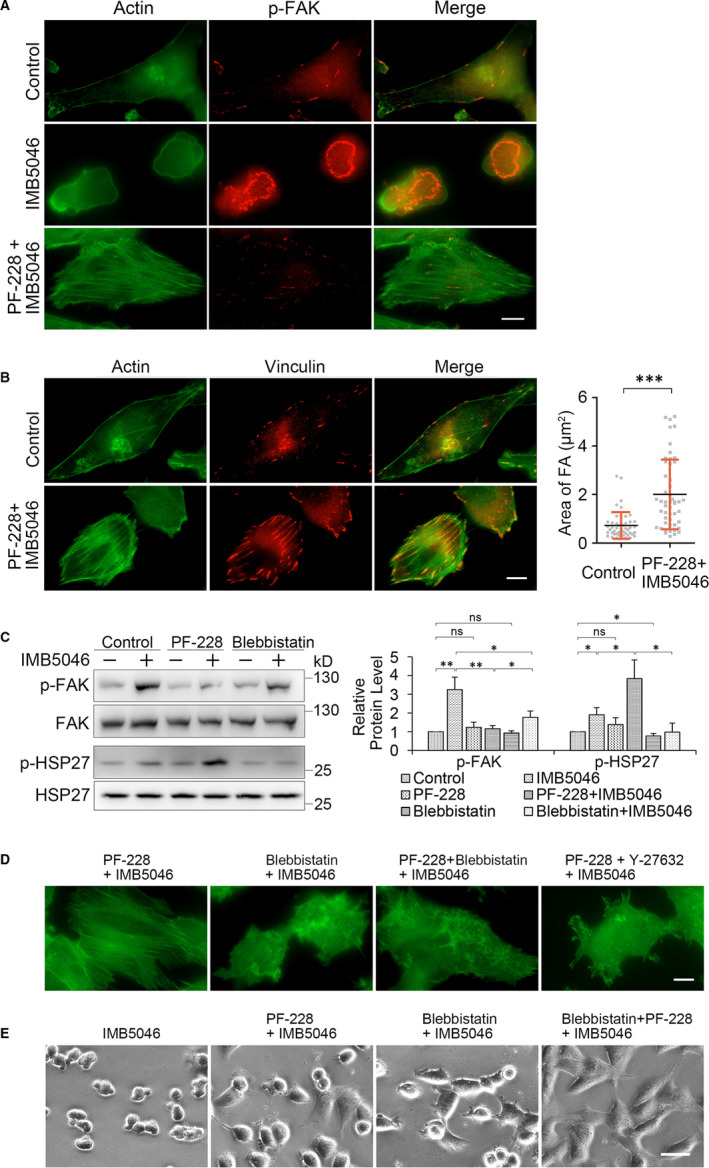
IMB5046 stimulates the formation of stress fibres in cells pre‐treated with PF‐228 via promoting the phosphorylation of HSP27. A, IMB5046 induces the stress fibre formation in cells pre‐treated with PF‐228. HMEC‐1 cells were pre‐treated with PF‐228 (10 μM, 30 minutes) or not, then exposed to 1 μM IMB5046 for 1 hour. Cells were stained with phalloidin‐FITC and p‐FAK antibody. For IMB5046‐treated cells, left panel, focal plane at the middle of the cells; middle panel, focal plane at the basal side of the cells. Bar, 10 μm. B, HMEC‐1 cells were pre‐treated as above, then stained with phalloidin‐FITC and vinculin antibody. The area of FAs was calculated according to the vinculin staining. Data are presented as mean ± SD (n = 50). Bar, 10 μm. C, IMB5046 promotes the phosphorylation of HSP27 in cells pre‐treated with PF‐228. HMEC‐1 cells were pre‐treated with 10 μM PF‐228 or blebbistatin for 30 minutes, then exposed to 1 μM IMB5046 for 1 hour. Representative images of Western blot are shown. The histogram shows the relative protein level. Data are presented as mean ± SD (n = 3). D, Regulation of stress fibres formation in IMB5046‐treated cells. HMEC‐1 cells were pre‐treated with different inhibitors (10 μM) or its combination for 30 minutes, then exposed to 1 μM IMB5046 for 1 hour. Cells were stained with phalloidin‐FITC. Bar, 10 μm. E, Regulation of cell morphology in IMB5046‐treated cells. HMEC‐1 cells were pre‐treated with different inhibitors (10 μM) or its combination for 30 minutes, then exposed to 1 μM IMB5046 for 1 hour. Bar, 40 μm. ***P < .001. **P < .01. *P < .05. ns, no significance

Then, we want to know whether pre‐treatment with FAK inhibitor could prevent IMB5046‐induced blebbing. The result showed that in PF‐228‐pre‐treated cells, IMB5046 partially induced cell contraction, whereas failed to induce blebbing. After exposure to IMB5046 for 1 hour, about 62.2% cells showed strong cytoplasmic stress fibres (Figure 5A), compared with 6.2% in the IMB5046‐treated cells. A thin layer of actin cortex was formed at the dorsal side of the cells, and a strong staining of F‐actin was observed at the ventral side of the cells (Figure 1C). According to vinculin staining, the size of FAs increased about 4 times compared with that of the control cells (Figure 5B). PF‐228 alone did not induce the formation of stress fibres or increase the size of FAs (Figure S3). Though blebbistatin also inhibited blebbing, in the blebbistatin‐pre‐treated cells, IMB5046 did not induce the formation of stress fibres (Figure 5D, Blebbistatin + IMB5046). It indicates that PF‐228 and blebbistatin show different inhibitory mechanism.

The level of p‐HSP27 is highly related with the actin polymerization. Western blot assay showed that in PF‐228‐pre‐treated cells, IMB5046 significantly promoted the phosphorylation of HSP27, whereas not in blebbistatin‐pre‐treated cells (Figure 5C). Compared with IMB5046‐treated cells, both PF‐228 and blebbistatin pre‐treatment blocked IMB5046‐stimulated phosphorylation of FAK, whereas PF‐228 blocked it more obviously.

To investigate whether myosin or ROCK takes part in the formation of stress fibres, cells were pre‐treated with different inhibitors or their combinations for 30 minutes; then, IMB5046 was added. The result showed that IMB5046 stimulated the formation of a large number of stress fibres in PF‐228‐pre‐treated cells (Figure 5D, PF‐228 + IMB5046), whereas not in cells pre‐treated with PF‐228 plus blebbistatin (Figure 5D, PF‐228 + Blebbistatin + IMB5046) or PF‐228 plus Y‐27632 (Figure 5D, PF‐228 + Y27632 + IMB5046). It demonstrates that both actomyosin contractility and ROCK activity are needed for the formation of stress fibres.

Then, the effect of different inhibitors on IMB5046‐induced morphology changes was investigated. Consistent with previous observation, IMB5046 induced contraction of HMEC‐1 cells (Figure 5E, IMB5046). Though both PF‐228 (Figure 5E, PF‐228 + IMB5046) and blebbistatin (Figure 5E, Blebbistatin + IMB5046) partially inhibited the morphology changes induced by IMB5046, when PF‐228 and blebbistatin were used together (Figure 5E, Blebbistatin + PF‐228 + IMB5046), the morphology changes were completely inhibited. Y‐27632 also completely inhibited IMB5046‐induced morphology changes (Figure S4). These results demonstrate that both the disassembly of FAs and myosin contraction are needed for IMB5046‐induced morphology changes, and Rho/ROCK pathway localizes at the upstream of MLC2 and FAK.

## 4 DISCUSSION

MDAs can be used as vascular disrupting drugs.^15^ As a newly discovered MDA, we first study the effects of IMB5046 on human endothelial cells. We found that IMB5046 and many other MDAs could induce endothelial blebbing, whereas the microtubule‐stabilizing agents did not. Contraction and blebbing of endothelial cells may cause narrowing of the vessels and increase vascular resistance, and finally impair the function of vessels. This result provides a possible mechanism for MDAs, but not microtubule‐stabilizing agents, to be used as vascular disrupting drugs. We also found that IMB5046 selectively induced blebbing of endothelial cells, whereas not of NIH/3T3 or NCI‐H460 cells. Blebbing is a dynamic process highly related to actin cortex and regulated by many proteins such as Rho GTPases.^27^ This selectivity may be due to the differential expression of these proteins. In our experiments, knock‐down expression of FAK or GEF‐H1 in HMEC‐1 cells abolished IMB5046‐induced blebbing. In the future study, the mechanism of this selectivity will be further studied.

IMB5046 can induce reassembly of FAs and activation of FAK. Treatment with IMB5046 decreased the size of FAs, whereas pre‐treatment with FAK inhibitor PF‐228 enlarged them. This result is consistent with previous report that cells from FAK‐deficient mice showed enhanced FA formation.^13^, ^28^ It also verifies that activation of FAK can regulate FA dynamics and disassembly.^29^

**Figure 6:**
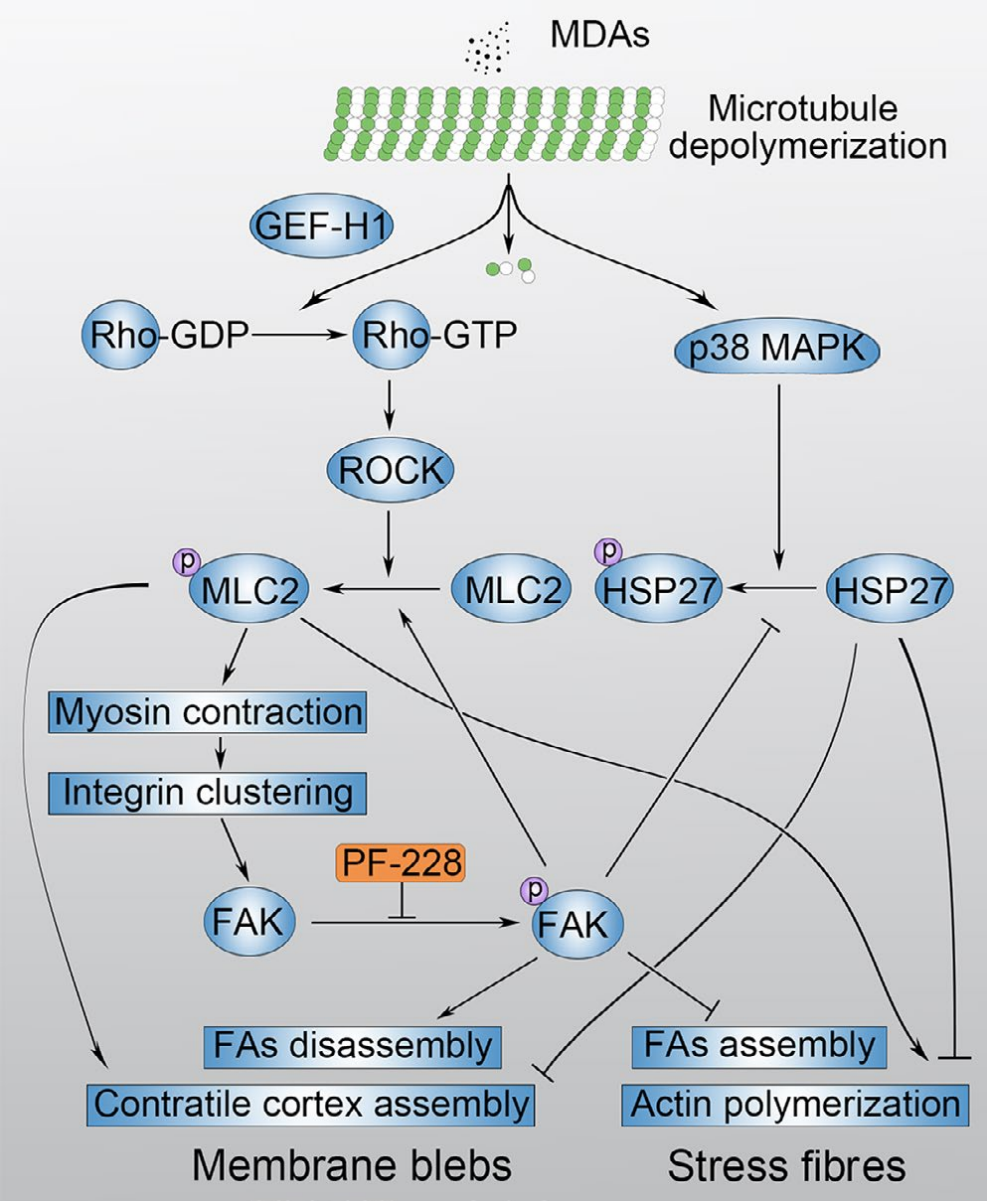
A proposed model of MDA‐induced activation of FAK and the regulation of blebbing and stress fibre formation. MDAs induce the activation of FAK via GEF‐H1/ Rho/ ROCK/ MLC2/ integrin pathway. After activation, FAK further promotes the phosphorylation of MLC2 and causes more intensive actomyosin contraction and continuous membrane blebbing. FAK inhibitor blocks membrane blebbing via inhibiting actomyosin contraction, and stimulated stress fibre formation via promoting the phosphorylation of HSP27

IMB5046 stimulated activation of FAK and MLC2. It has been reported that CA‐4‐P stimulates phosphorylation of MLC2 via Rho/ROCK pathway.^16^ Rho/ROCK pathway is also critical to FAK activation by cyclic stretch in cardiac myocytes.^30^ However, whether MDAs stimulate phosphorylation of FAK via Rho/ROCK pathway is unclear. Our experiments proved that IMB5046 induced the activation of FAK via Rho/ ROCK/ MLC2/ integrin pathway (Figure 6). ROCK can phosphorylate MLC2 directly and/or inhibit myosin phosphatase to promote MLC2 phosphorylation,^23^, ^31^ which leads to formation of myosin filament and interaction of actin and myosin. The tension exerted on the actin filaments will be transmitted to the integrins to which they are attached, leading to integrin aggregation.^24^ Aggregation of integrins will stimulate autophosphorylation and activation of FAK.^24^ Our experiment also proved that binding of integrins with extracellular matrix was required for blebbing. As inhibiting the activity of myosin phosphatase may contribute to IMB5046‐induced myosin contraction, it is possible that phosphatase inhibitors will further inhibit its activity and promote MDA‐induced blebbing. Next question is how microtubule deploymerization activates Rho. Result of RNAi demonstrated that GEF‐H1 was involved in blebbing. Another guanine nucleotide exchange factor p190RhoGEF can also bind with both microtubule and FAK.^32^, ^33^, ^34^ Whether p190RhoGEF plays a role in the blebbing process needs to be further studied.

IMB5046 stimulated phosphorylation of MLC2 and finally induced FAK activation. We also found that FAK inhibitor down‐regulated the phosphorylation of MLC2. It indicates that FAK positively regulates the phosphorylation of MLC2. We propose that this positive feedback would cause more intensive cortex contraction and continuous membrane blebbing (Figure 6). FAK can regulate the activity of Rho through the selective association with p190RhoGEF or p190ARhoGAP, leading to activation or inactivation of ROCK and MLC2.^12^ Our experiment proved that FAK inhibitor showed no obvious effect on Rho activity stimulated by IMB5046. Thus, FAK may exert its effect on ROCK or MLC2 via other pathway. Besides activated by Rho, ROCK can also be activated by caspase‐3 or granzyme‐mediated C‐terminus cleavage.^35^ The effect of FAK inhibition on ROCK activity needs to be further studied. The activity of MLC2 is regulated by the balance of MLCK and myosin phosphatase. FAK‐Src signalling can promote the phosphorylation of MLCK via ERK,^13^ whereas MLCK inhibitor ML‐7 and ML‐9 showed no effect on IMB5046‐induced blebbing. It is reported that integrin‐linked kinase (ILK) and zipper‐interacting protein kinase (ZIPK) could inhibit the activity of myosin phosphatase and phosphorylate MLC2 directly.^36^ It is possible that FAK activates MLC2 via ILK or ZIPK. Another possible pathway is that FAK activates MLC2 via PKC/ CPI‐17/ myosin phosphatase pathway.^37^, ^38^ In addition, CPI‐17 can also be phosphorylated by ROCK, ILK and ZIPK directly.^36^ The effect of FAK inhibition on these pathways will be studied in the future.

In our study, no matter PF‐228 was given before or after IMB5046, it abolished IMB5046‐induced blebbing. We also tried to give IMB5046 and PF‐228 simultaneously. In this condition, IMB5046 failed to induce blebbing, whereas the cells contracted more intensively compared with cells pre‐treated with PF‐228 (data not shown). Many reports show that microtubule disassembly results in the activation of Rho, which enhances myosin contractility and stress fibre formation.^16^, ^39^, ^40^, ^41^ In our experiment, IMB5046 stimulated activation of Rho, myosin contraction and blebbing, whereas failed to stimulate stress fibre formation. When FAK activity was inhibited by PF‐228 or FAK expression was down‐regulated by RNAi, IMB5046 stimulated stress fibre formation. Mechanism study revealed that under condition FAK activity was inhibited, IMB5046 induced high level of phosphorylated HSP27. It indicates that FAK negatively regulates the phosphorylation of HSP27 and inhibits the formation of stress fibres (Figure 6). HSP27 is located at the downstream of p38 MAPK/MAPK‐activated protein kinase 2 (MAPKAPK2) pathway.^42^ As IMB5046 induced the phosphorylation of HSP27 and p38 MAPK inhibitor SB203580 abolished IMB5046‐induced blebbing (Table S1), we propose that IMB5046 induces the activation of p38 MAPK/MAPKAPK2/HSP27 pathway. A relative low level of phosphorylated HSP27 is needed for blebbing, whereas high level of phosphorylated HSP27 is needed for stress fibre formation.

In summary, IMB5046 and many other MDAs can induce endothelial blebbing. FAK is activated by MDAs via GEF‐H1/ Rho/ ROCK/ MLC2/ integrin pathway, and it is a molecular switch controlling membrane blebbing and stress fibre formation.

## CONFLICT OF INTEREST

The authors confirm that there are no conflicts of interest.

## AUTHOR CONTRIBUTIONS

Yan‐Bo Zheng designed and performed the experiments, analysed the data and wrote the manuscript. Jian‐Hua Gong performed the experiments, analysed the data and revised the manuscript. Yong‐Su Zhen supervised the work and revised the manuscript. All authors read and approved the final manuscript.

## Supporting information

Supplementary Material

Video S1

Video S2

## Data Availability

The datasets used in the current study are available from the corresponding author on reasonable request.
