# Supplementary material for: Focal adhesion kinase is activated by microtubule‐depolymerizing agents and regulates membrane blebbing in human endothelial cells
Source: J Cell Mol Med. 2020 May 26;24(13):7228–38. doi: 10.1111/jcmm.15273 (PMC7339229; doi:10.1111/jcmm.15273)
Supplement: Supplementary file 1 — Supplementary Material [file JCMM-24-7228-s001.docx]

**Supplementary files**

**
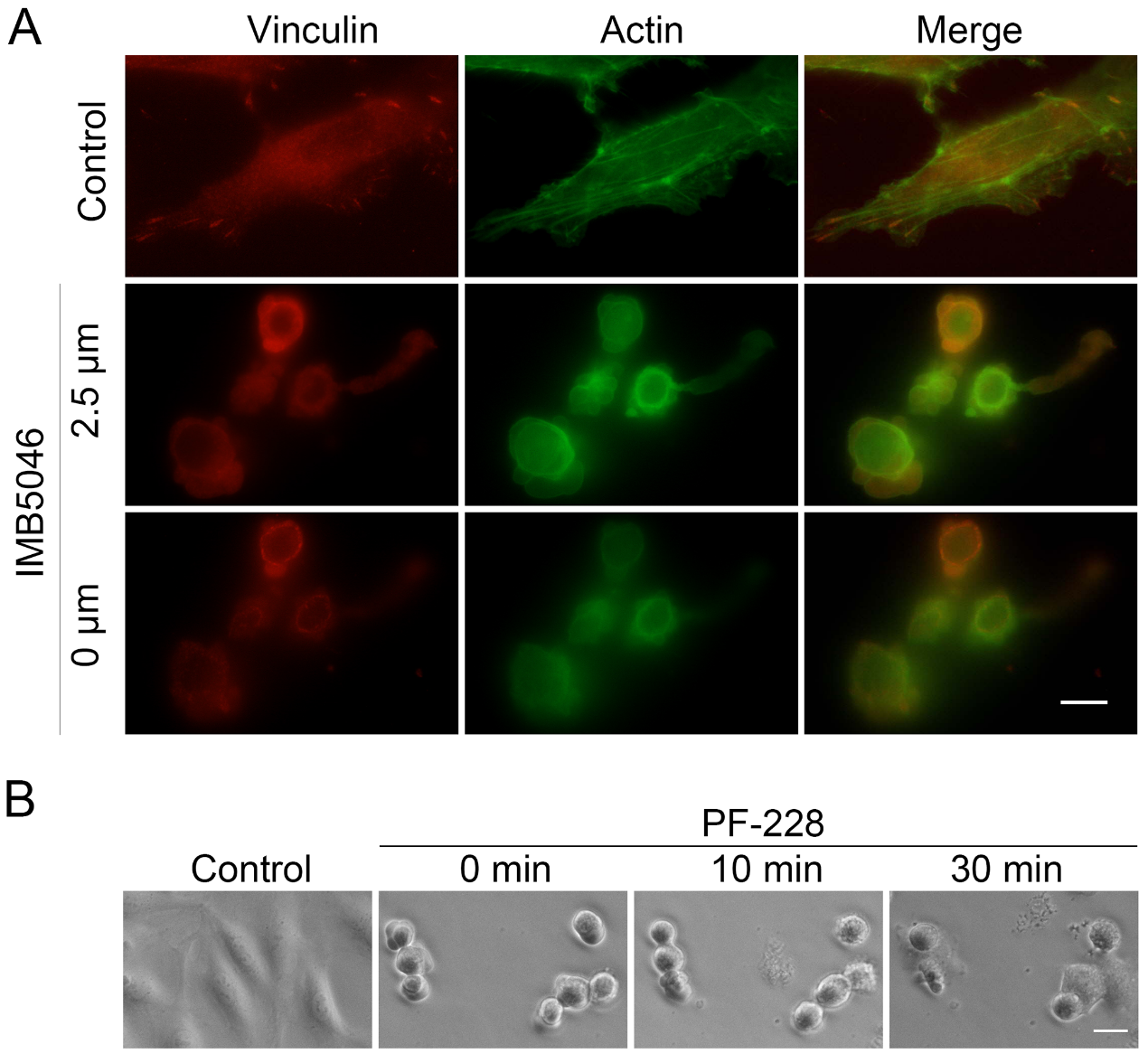
**

**FIGURE S1** IMB5046 induces blebbing in HUVECs and PF-228 blocks it. A, HUVECs were treated with 1 μM IMB5046 for 1 h, then stained with vinculin antibody and phalloidin-FITC. 2.5 μm, focal plane 2.5 μm above the bottom. 0 μm, focal plane 0 μm above the bottom. Bar, 10 μm. B, HUVECs were treated with 1 μM IMB5046 for 1 h, then exposed to 10 μM PF-228 for indicated time. Bar, 20 μm.


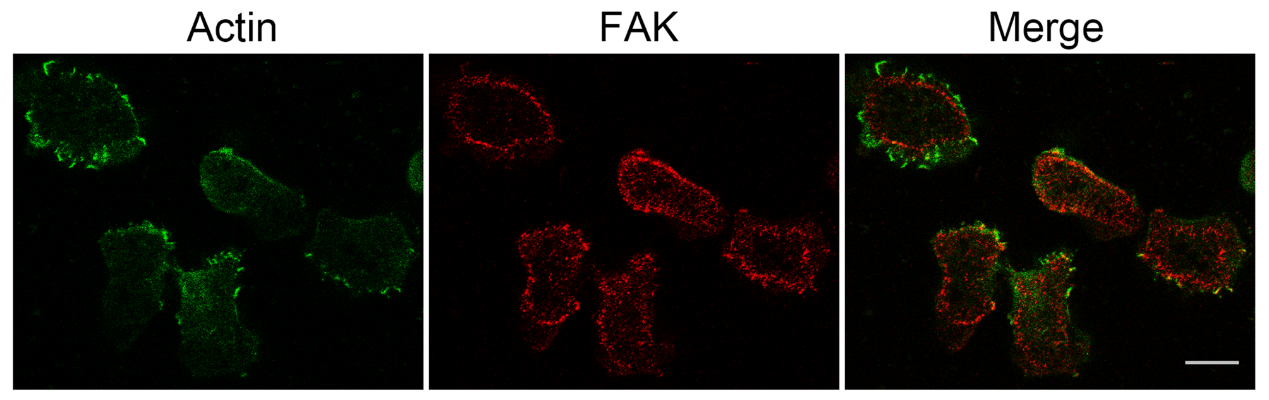


**FIGURE S2** FAK partially co-localizes with actin cortex. HMEC-1 cells were exposed to 1 μM IMB5046 for 1 h, then stained with phalloidin-FITC and FAK antibody. Images were taken by laser confocal microscopy. Bar, 10 μm.


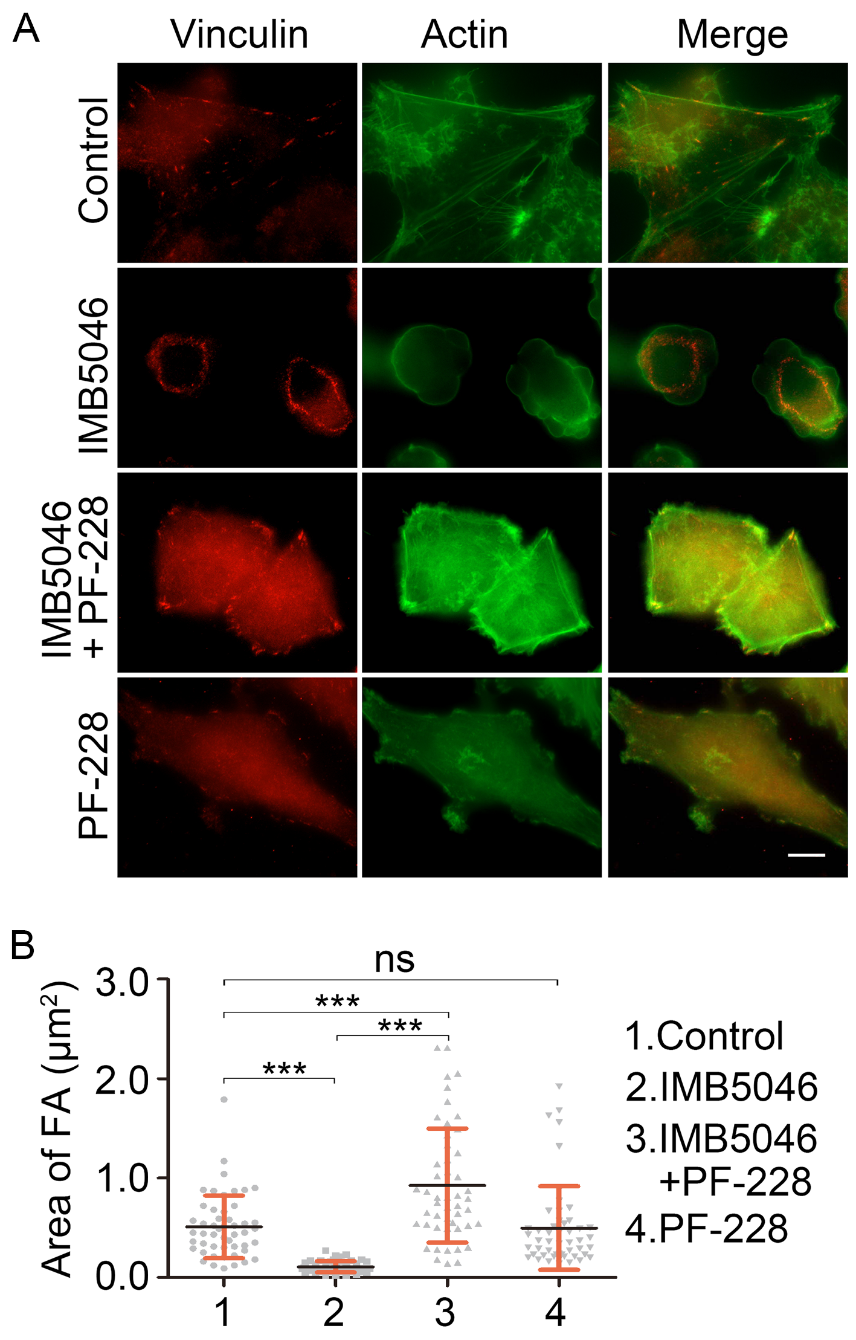


**FIGURE S3** PF-228 blocks IMB5046-induced blebbing. A, HMEC-1 cells were treated with 1 μM IMB5046 for 1 h, then exposed to PF-228 (10 μM, 30 min) or not. Cells were stained with vinculin antibody and phalloidin-FITC. For IMB5046-treated cells, non-processed images from basal and midsection focal planes show FAs and membrane blebs, respectively. For other groups, non-processed images were taken from the same focal planes at basal side of the cells. Bar, 10 μm. B, Area of FAs were calculated according to the vinculin staining. Data are presented as mean ± SD (n = 50). *** P < 0.001. ns, no significance.

**

**

**Figure S4** Y-27632 inhibits the morphology changes induced by IMB5046. HMEC-1 cells were pretreated with 10 μM Y-27632 for 30 min, then exposed to 1 μM IMB5046 for 1 h. Bar, 50 μm.

**MOVIE S1** IMB5046 induces membrane blebbing of HMEC-1 cells. HMEC-1 cells were treated with 1 μM IMB5046 for 1 h, then observed using live-cell imaging. Frames were taken every 5 s.

**MOVIE S2** PF-228 inhibits IMB5046-induced membrane blebbing. HMEC-1 cells were treated with 1 μM IMB5046 for 1 h, then exposed to 10 μM PF-228. Timing relative to PF-228 exposure is indicated. Frames were taken every 5 s.

**Table S1** Effects of different inhibitors on IMB5046-induced membrane blebbing.

| Inhibitors | Target | Inhibit blebbing? |
| --- | --- | --- |
| PF-228, PF-431396, TAE226 | FAK | Yes |
| Dasatinib, Bosutinib | Src | Yes |
| C3 exoenzyme | Rho | Yes |
| EHop-016 | Rac | No |
| ML141 | CDC42 | No |
| Y-27632 | ROCK | Yes |
| ML-7, ML-9 | MLCK | No |
| Blebbistatin | Myosin II | Yes |
| cRGD | Integrin | Yes |
| SB203580 | p38 MAPK | Yes |
| HMEC-1 cells were pretreated with C3 exoenzyme (1 μg/mL, 6 h), then exposed to 1 μM IMB5046 and observed using live-cell image. For all other inhibitors, HMEC-1 cells were treated with IMB5046 (1 μM, 1h) to induce blebbing, then different inhibitors (10 μM) were added and observed using live-cell image. | | |
